# A Splice Site Variant in the Bovine *RNF11* Gene Compromises Growth and Regulation of the Inflammatory Response

**DOI:** 10.1371/journal.pgen.1002581

**Published:** 2012-03-15

**Authors:** Arnaud Sartelet, Tom Druet, Charles Michaux, Corinne Fasquelle, Sarah Géron, Nico Tamma, Zhiyan Zhang, Wouter Coppieters, Michel Georges, Carole Charlier

**Affiliations:** 1 Unit of Animal Genomics, GIGA-R and Department of Animal Sciences, Faculty of Veterinary Medicine, University of Liège, Liège, Belgium; 2 Unit of Bioinformatics, Department of Animal Sciences, Faculty of Veterinary Medicine, University of Liège, Liège, Belgium; University of Illinois at Urbana-Champaign, United States of America

## Abstract

We report association mapping of a locus on bovine chromosome 3 that underlies a Mendelian form of stunted growth in Belgian Blue Cattle (BBC). By resequencing positional candidates, we identify the causative c124-2A>G splice variant in intron 1 of the *RNF11* gene, for which all affected animals are homozygous. We make the remarkable observation that 26% of healthy Belgian Blue animals carry the corresponding variant. We demonstrate in a prospective study design that approximately one third of homozygous mutants die prematurely with major inflammatory lesions, hence explaining the rarity of growth-stunted animals despite the high frequency of carriers. We provide preliminary evidence that heterozygous advantage for an as of yet unidentified phenotype may have caused a selective sweep accounting for the high frequency of the *RNF11* c124-2A>G mutation in Belgian Blue Cattle.

## Author Summary

Recessive defects in livestock are common, and this is considered to result from the contraction of the effective population size that accompanies intense selection for desired traits, especially when relying heavily on artificial insemination (as males may concomitantly have a very large number of offspring). The costs of recessive defects are assumed to correspond to the loss of the affected animals. By performing a molecular genetic analysis of stunted growth in Belgian Blue Cattle (BBC), we highlight (i) that the economic impact of recessive defects may outweigh the only loss of affected animals and (ii) that some genetic defects are common for reasons other than inbreeding. We first demonstrate that a splice site variant in the *RING finger protein 11* (*RNF11*) gene accounts for ∼40% of cases of stunted growth in BBC. We then show that a large proportion of animals that are homozygous for the corresponding *RNF11* mutation die at a young age due to compromised resistance to pathogens. We finally demonstrate that carriers of the mutation benefit from a selective advantage of unidentified origin that accounts for its high frequency in BBC.

## Introduction

Growth is one of the economically most important phenotypes in livestock production. While genetic variants with large effects on stature account for part of the between-breed variation [1], within-breed variation is likely to be highly multifactorial and polygenic. Accordingly, quantitative trait loci (QTL) influencing growth are reported on all autosomes in the cattle QTL database (http://www.animalgenome.org/cgi-bin/QTLdb/BT/index).

The BBC breed is a beef breed that is famous for its “double-muscling” phenotype caused in part by a disruptive 11-bp deletion in the myostatin (*MSTN*) gene [2]. As in other breeds, growth performances are paramount in BBC as they control duration of the fattening period and final carcass weight, hence directly determining profit.

In recent years, an increasing number of young animals with growth retardation as primary symptoms were reported to our heredosurveillance platform. We established this platform in 2005 to rapidly detect genetic defects emerging in the BBC, identify the culprit genes and mutations, and develop diagnostic tests to limit their negative impact [3]. Animals with growth retardation underwent a standard protocol including a genome-wide association study (GWAS) to identify putative causative loci. We herein report the mapping of a locus accounting for ∼40% of growth-retardation cases, and identify the causative loss-of-function mutation in the *RING finger protein 11* (*RNF11*) gene. Moreover, we perform a prospective study that indicates that as much as one third of homozygous mutants die from infection before six months of age. We finally present evidence that carriers of the mutation might benefit from a selective advantage that may account for its unexpectedly high frequency (∼13%) in the BBC population.

## Results

### A major growth-stunting locus maps to BTA3

**Figure 1:**
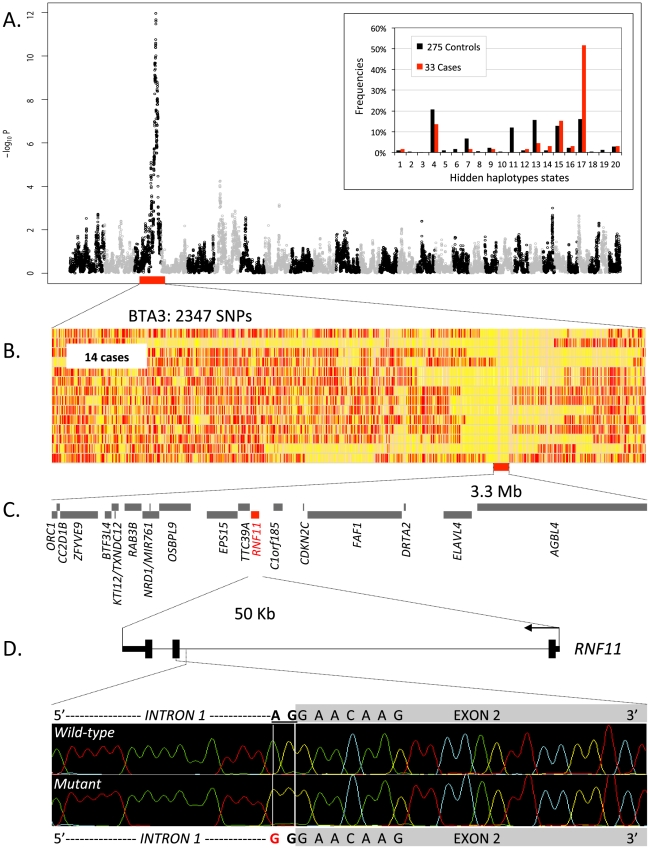
Genome-wide haplotype-based association mapping of a growth stunting locus on BTA 3. (A) Manhattan plot for the haplotype-based genome-wide association study for stunted growth using a model with 20 ancestral haplotypes. Alternating colors (black and grey dots) mark the limits between autosomes. Inset: frequency of the 20 hidden haplotype states in the 33 cases (red) and the 275 controls (black) at position BTA3:103,391,968 bp. (B) Genotypes of the 14 cases homozygous for hidden haplotype state 17 for 2,347 BTA3 SNPs. Homozygous genotypes are shown in orange or yellow and heterozygous genotypes in red. The limit of the homozygous haplotype shared by the 14 cases is highlighted in red. (C) Gene content of the 3.3 Mb shared interval (19 genes). (D) *RNF11* gene model, and representation of the *RNF11* c124-2A>G splice site variant.

Between 2008 and 2011, we collected blood samples and epidemiological data from 147 BBC individuals, aged between 3 months and 3 years old, with pronounced (∼15% reduction in stature when compared to contemporaries) yet proportionate growth retardation as primary distinctive feature. We initially genotyped 33 of these with a custom-designed 50 K medium-density bovine SNP array [3]. None of these animals would be homozygous or compound heterozygote for the previously identified c.2904-2905delAG [4] and c.1906T>C [5] *MRC2* mutations causing Crooked Tail Syndrome and known to affect stature. Using the genotypes of the corresponding SNPs (yet obtained with a distinct, high-density bovine SNP array) from 275 healthy sires as control, we performed a GWAS using an approach based on hidden haplotype states with a generalized mixed model accounting for stratification (Zhang *et al.*, submitted for publication). A genome-wide significant signal was obtained on BTA3 driven by haplotype state 17, observed at a frequency of 52% in cases versus 12% in controls (Figure 1A). Fourteen of the 33 cases (42%) were homozygous for the corresponding haplotype, causing a significant deviation from Hardy-Weinberg expectations in cases (expected: 27%, p<0.002), hence suggesting recessivity.

**Figure 2:**
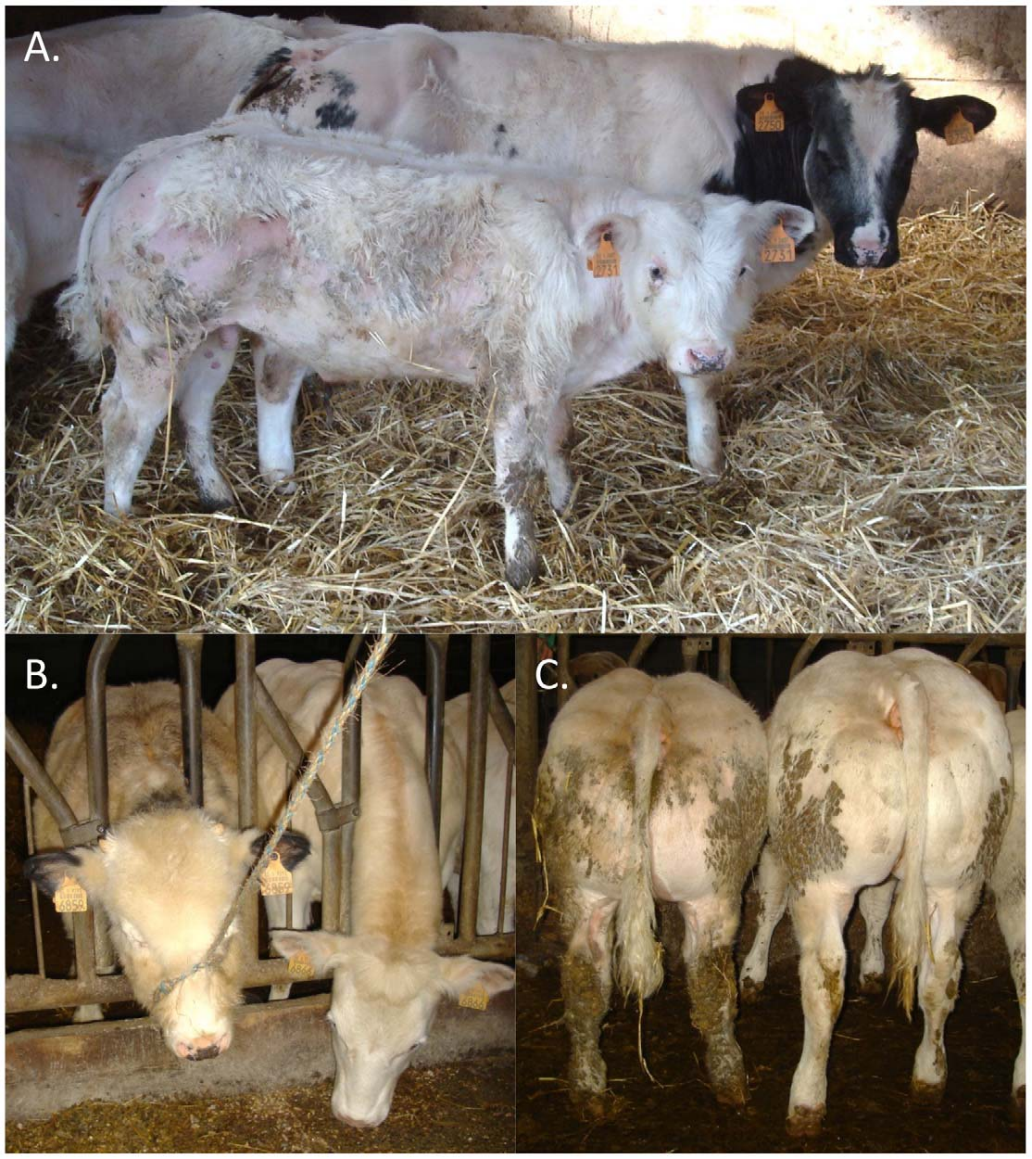
Features of animals homozygous for the *RNF11* c124-2A>G mutation. Affected (front) and control (back) calves of same age, illustrating the proportionate growth retardation, close forehand, and hairy head masking a narrow skull (A). Illustration of the hairy head (B), and normal muscle development (C) of animals homozygous for the *RNF11* c124-2A>G variant.

Retrospective phenotypic analysis of the 14 homozygotes revealed shared features: proportionate growth retardation appearing around 5–6 months of age (not observed at birth), normal muscular development, close forehand, long and thin neck, hairy, long and thin head (Figure 2). Pedigree analysis indicated that the 14 individuals traced back to *Galopeur des Hayons* (a once popular BBC sire) on sire and dam side.

### A splice site mutation in the *RNF11* gene is the likely causative mutation

Direct examination of the SNP genotypes of the 14 cases homozygous for hidden state 17 revealed a 3.3 Mb (100,727,788–104,017,608 - Btau 4.0) segment of autozygosity (Figure 1B). It encompassed 19 annotated genes of which none was an obvious candidate (Figure 1C). We thus undertook the systematic re-sequencing of all open reading frames (ORF) and intron-exon boundaries. During this process (and after completion of 14/19 genes), we identified an A to G transition (c124-2A>G) mutating the intron 1 acceptor splice site of the *RNF11* gene (Figure 1D). *RNF11* encodes a highly conserved, ubiquitously expressed protein with 154 amino-acids [6], recently recognized as a subunit of the A20 ubiquitin-editing complex regulating NF-κβ signaling [7]. We developed a 5′-exonuclease assay and genotyped (i) the case-control cohort used for GWAS (33 cases, 275 controls), (ii) a diversity panel encompassing 141 animals from eleven breeds other than BBC, (iii) 549 additional normal adult BBC animals, and (iv) *Galopeur des Hayons*. The c124-2A>G variant appeared in near perfect linkage disequilibrium (D′ = 1; r^2^ = 0.984) with haplotype state 17 in the case-control cohort. It was not present in non-BBC animals. It had an allelic frequency of 13% amongst the 824 genotyped healthy adult BBC animals, yet without a single animal being homozygous *GG* (p<0.01 under Hardy-Weinberg equilibrium). *Galopeur* was indeed confirmed to be carrier of the c124-2A>G mutation.

**Figure 3:**
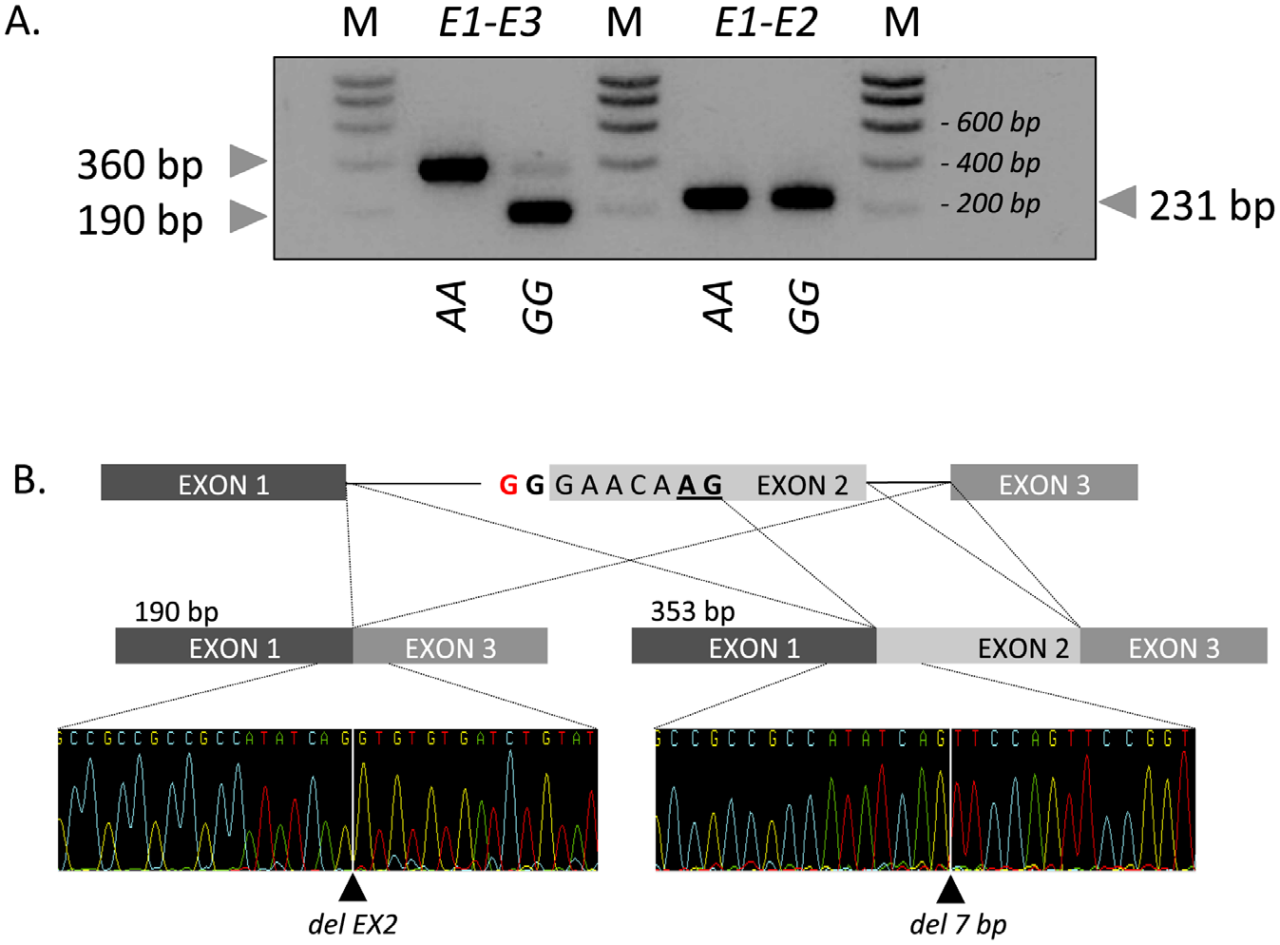
Effect of the c124-2A>G splice site variant on *RNF11* transcripts. (A) Gel electrophoresis of RT-PCR products obtained from mesenteric lymph node from homozygous wild-type (*AA*) and mutant (*GG*) animals using primer sets located respectively in exon 1 and 3 (E1–E3) and exon 1 and 2 (E1–E2). M: molecular weight marker. (B) Sequence analysis and structure of the 190-bp and 360-bp RT-PCR products obtained from an affected (*GG*) animal.

The effect of the c124-2A>G mutation on *RNF11* transcripts was examined by RT-PCR using RNA extracted from skeletal muscle, spleen, mesenteric lymph node, thymus, lung, trachea of one *GG* and one *AA* animal. Using two primers located respectively in exon 1 and 3 and RNA from wild-type *AA* animals, we obtained a unique 360-bp RT-PCR product in all examined tissues, and showed by sequencing that it encompassed the expected exon 2 sequence (data not shown). The same experiment performed with RNA from a homozygous mutant *GG* animal yielded (i) a major product of ∼190 bp, and (ii) a minor product of ∼360 bp (Figure 3A). The major product was shown by sequencing to correspond to a transcript skipping exon 2. The minor product missed the first seven base pairs of exon 2, and resulted from the activation of a cryptic splice site in exon 2. RT-PCR conducted with primers located respectively in exon 1 and 2 confirmed the existence of transcripts containing exon 2 in homozygous mutants (Figure 3B). Both forms are expected to cause a frameshift, appending 29 (major product) and 14 (minor product) illegitimate residues to a severely truncated (41/154 amino-acids) RNF11 protein missing the ubiquitin interaction and RING-finger domains. The transcript corresponding to the minor form is expected to undergo non-sense mediated RNA decay (NMRD) [8], due to the occurrence of a stop codon in exon 2 of three. NMRD is not expected to affect the transcript corresponding to the major form as the corresponding open reading frame terminates in exon 3 of three. We compared the levels of *RNF11* transcript in mesenteric lymph node and spleen of a wild-type *AA* and a mutant *GG* animals, using quantitative RT-PCR with primer sets targeting the second (outside of the 7-bp deletion) and third *RNF11* exons, respectively, as well as three internal control genes. In spleen, we observed a 1.1-fold reduction (p = 0.4) in the amount of exon 3 containing transcripts, and a 11-fold reduction (p<0.005) in exon 2 containing transcripts. Assuming NMRD of the minor but not of the major product, this allows us to estimate (i) that ∼80% of the *RNF11* pre-mRNAs skip exon 2, while ∼20% use the exon 2 cryptic splice site, and (ii) that 55% of exon 2 retaining transcripts are being degraded by NMRD. The same analysis conducted in lymph node reveals a ∼2-fold reduction (p<0.05) in exon 3 containing transcripts, and ∼37-fold reduction (p<0.0005) in exon 2 containing transcripts, corresponding to (i) ∼44% of *RNF11* pre-mRNAs skipping exon 2 and ∼56% using the exon 2 cryptic splice site, and (ii) ∼95% of exon 2 retaining transcripts being degraded by NMRD (Supporting Information S1).

Taken together, our findings strongly support the causality of the c124A>G *RNF11* mutation in determining stunted growth in homozygous *GG* animals.

### Increased juvenile mortality accounts for incongruent carrier frequency and disease incidence

The ∼26% carrier frequency amongst healthy individuals is incompatible with the number of reported cases of stunted growth. As an example, ∼6% of offspring of known carrier bulls should be affected, and such high figures were never recorded. We reasoned that this lower than expected incidence of cases might reflect elimination of mutant animals either before or after birth. Embryonic mortality of homozygous mutant fetuses has been reported for deficiency in uridine monophosphate synthetase (DUMPS) [9], Complex Vertebral Malformation (CVM) [10], [11] and Brachyspina (BS) (Charlier *et al.*, submitted for publication).

To test these hypotheses we first examined field data and tested the effect of sire carrier status on (i) “non return (in oestrus) rate” of inseminated cows between 28 and 280 days after insemination, and (ii) rate of mortality, morbidity and culling of offspring between birth and 14 months of age [12]. Non-return rates tended to be slightly decreased when cows were inseminated with semen from carrier sires (i.e. reproductive failure increased), but the effect was not significant (p = 0.66). Mortality, morbidity and culling tended to be increased in offspring of carrier sires, but this effect was not significant either (p = 0.89) (Supporting Information S1).

**Figure 4:**
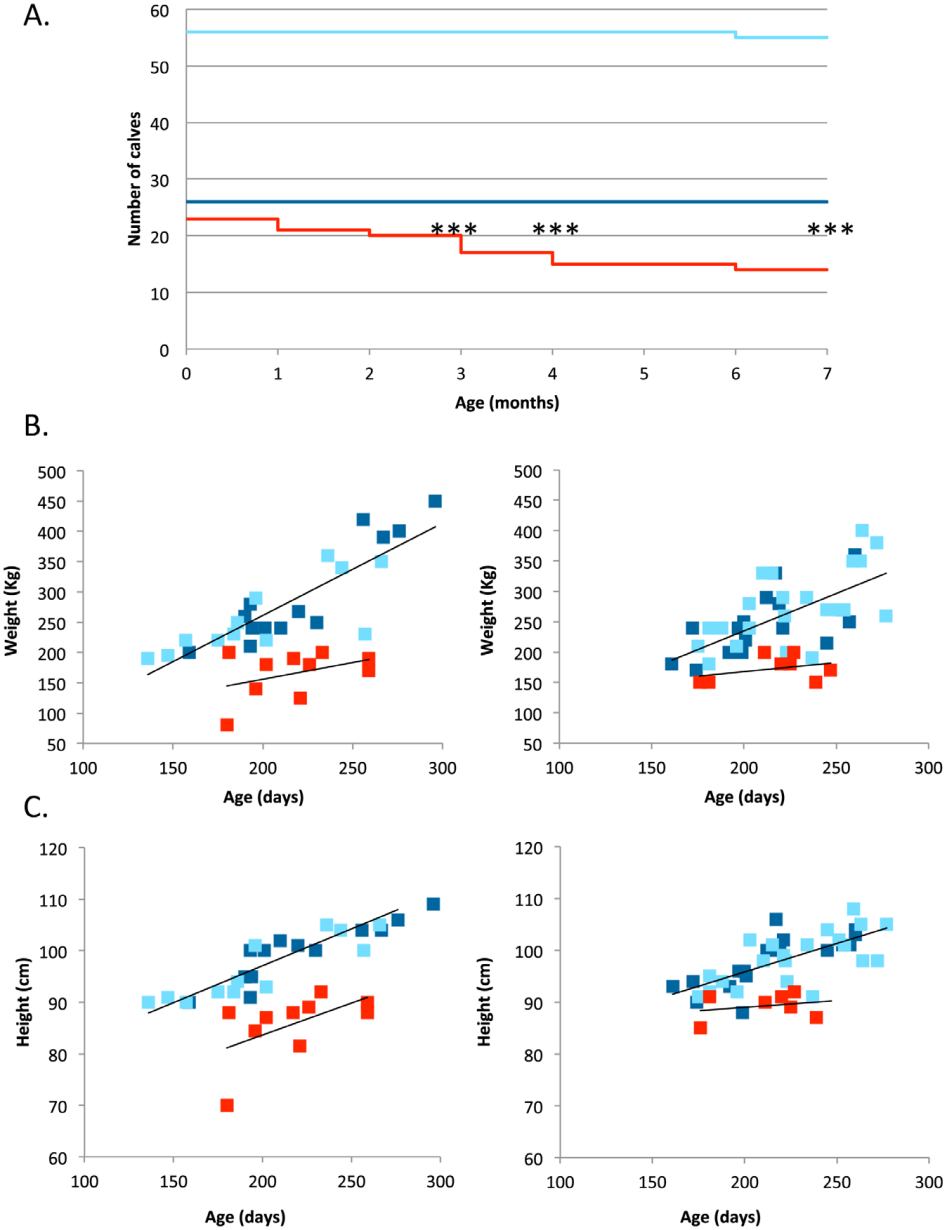
Survival and growth of 105 calves born from matings between carrier sires and dams. (A) Survival (from birth to 7 months of age) of calves sorted by c124-2A>G genotype (red: *GG*, dark blue: *AA*, light blue: *AG*) (***: p<0.001). (B) Weight (estimated from heart girth length) and (C) height at withers (from birth to 7 months of age) of calves sorted by c124-2A>G genotype (red: *GG*, dark blue: *AA*, light blue: *AG*). Regression lines (black) were fitted separately for affected and non-affected animals.

As analysis of field data did not provide conclusive results, we performed a prospective study. We identified 105 carrier dams in 22 farms that were pregnant following insemination with semen from known carrier sires. We followed the ensuing 105 calves up to 12 months after birth. The responsible veterinarian (AS) and the breeders were not aware of the calves' *RNF11* genotype until completion of the study. Genotypic proportions at birth did not deviate significantly from Mendelian expectations (*AA*: 26 ( = 24.8%); *AG*: 56 ( = 53.3%); *GG*: 23 ( = 21.9%); p = 0.72). All calves looked normal, and there was no significant effect of *RNF11* genotype on weight or height at birth. However, one year after birth, 10 calves had died and eight had been culled for health-related reasons. Strikingly, all but one of these were homozygous mutant *GG*, while one was *AG* (p<0.0005) (Figure 4A). While the *AG* animal was euthanized with a limb fracture, the nine deceased *GG* animals died with severe inflammation (primarily pneumonia) (Supporting Information S1). The c124-2A>G genotype had a highly significant (p≤0.001) effect on post-natal growth. Indeed, all surviving *GG* animals exhibiting stunted development after 6 months (Figure 4B). *A contrario*, the growth pattern of *AG* and *AA* animals was indistinguishable.

Taken together, our data indicate that as much as one third of homozygous *GG* calves die with major inflammation, while all remaining calves exhibit stunted growth and are hence systematically culled prematurely.

### Selective advantage of heterozygotes may underlie the high carrier incidence

The 26% carrier frequency amongst healthy BBC animals is puzzling given the observed purifying selection against *GG* animals. This suggests that heterozygotes might benefit from a selective advantage that would maintain the *G* allele at high frequency in the population. Such balanced polymorphism has been demonstrated for *MRC2* loss-of-function mutations causing Crooked Tail Syndrome in homozygotes, yet increased muscle mass in carriers [4], [5].

To test this hypothesis, we first used field data and examined the effect of *RNF11* c124-2A>G sire carrier status on own and progeny performances for recorded traits including size, muscularity, type and general appearance [12]. We obtained conflicting results: carrier status appeared to negatively affect the perceived quality of sire, yet improve the quality of its offspring (Supporting Information S1).

**Figure 5:**
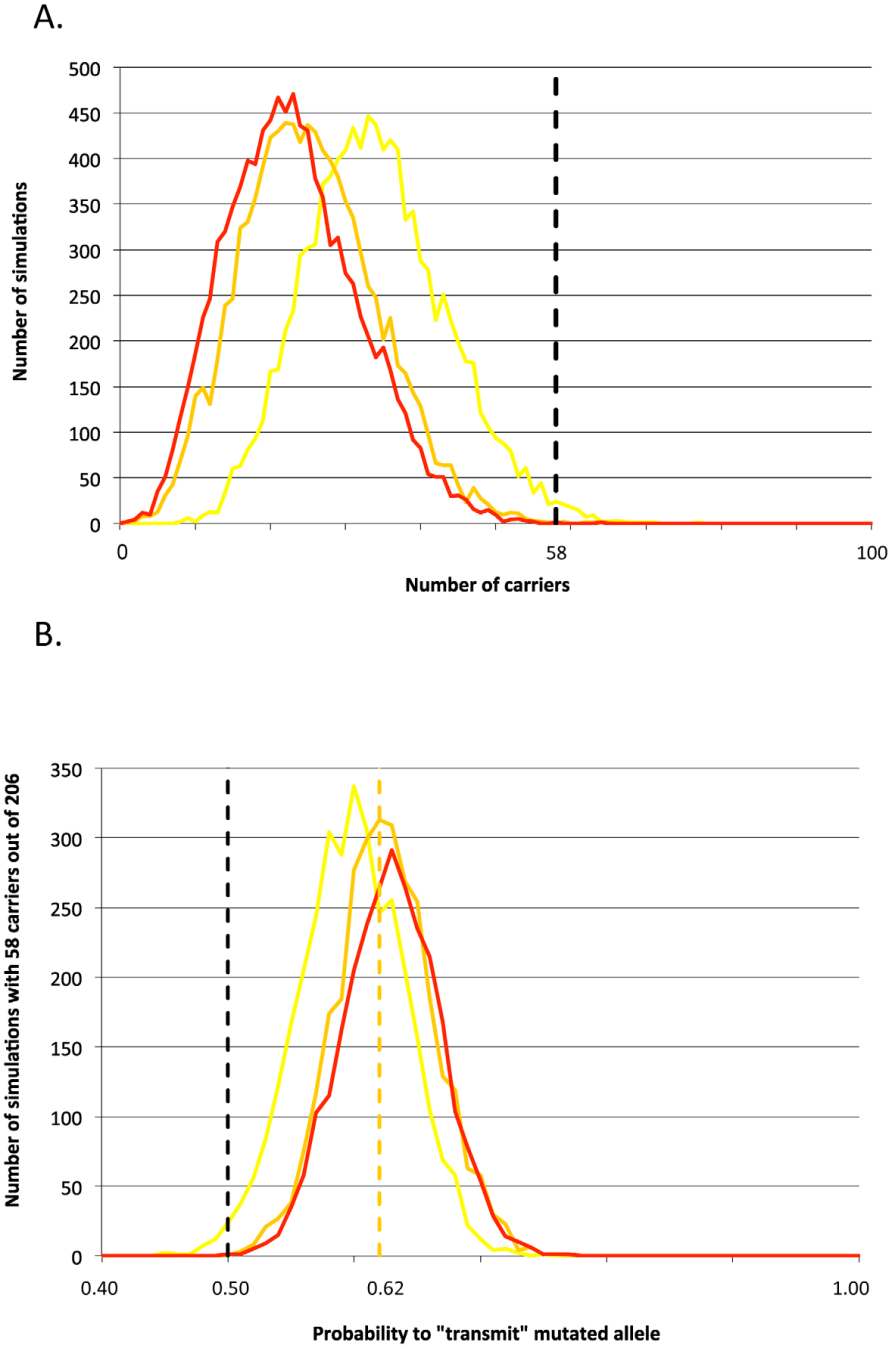
Signature of selection. (A) Frequency distribution (number of simulations out of 10,000) of the number of sires tracing back to the *Galopeur* founder (total: 206) that are expected to carry the c124-2A>G mutation assuming that it segregates in the corresponding pedigree according to Mendelian expectations, and that the frequency of c124-2A>G outside the *Gallopeur* lineage is 0% (red), 1% (orange), or 5% (yellow). The dotted vertical marks the actual number of carrier sires (58) amongst the 206 descendants of *Galopeur*. (B) Distribution of the number of simulations (out of 10,000) yielding 58 carriers out of 206 descendants of *Galopeur* (Y-axis), as a function of the rate of transmission of the mutation from heterozygous carriers (X-axis). Three curves are given corresponding to frequencies of the mutation outside of the Galopeur's lineage of 0% (red), 1% (orange), and 5% (yellow). The dotted orange vertical line corresponds to a transmission rate of 62%, maximizing the number of simulations yielding 58 carriers for a mutation frequency (outside of the Galopeur's lineage) of 1%.

As an alternative approach to test for a putative selective advantage benefitting carriers, we evaluated whether the incidence of carriers amongst active AI sires was compatible with Mendelian (0.5∶0.5) inheritance of a neutral mutation from the founder bull *Galopeur*. Assuming that the c124-2A>G mutation improves zootechnical performances in heterozygotes, carriers should be over-represented amongst AI sires related to *Galopeur*. Two hundred and six of the 262 BBC AI sires born between 2003 and 2007 were related to *Galopeur* and 58 ( = 28%) of these proved to carry the *RNF11* c124-2A>G mutation. Using gene dropping in the known genealogies, we computed the probability that 58 or more descendants would be carrier in the absence of selection (no systematic transmission distortion). This probability was 0.0002, 0.0006 and 0.01 assuming a frequency of 0, 0.01 and 0.05 for the c124-2A>G mutation outside the *Galopeur* lineage (Figure 5A). These results suggest that the c124-2A>G mutation indeed underwent a recent selective sweep in the BBC population, although the phenotype that is being selected remains unclear. That 58/206 descendents of *Galopeur* carry the c124-2A>G mutation is best explained by assuming that the mutation has ∼10% excess probability (i.e. 60%) to be transmitted by a carrier parent to an AI sire or one of its ancestors (Figure 5B).

### Lack of evidence for other major growth-stunting loci

Homozygosity at the *RNF11* c124-2A>G mutation accounted for 14 of the first 33 analyzed cases (i.e. 42%), raising the question of what caused stunted growth in the others. To address this, we genotyped the remaining 114 cases for the c124-2A>G mutation. In agreement with genotypic proportions in the first 33 cases, 47/114 (41%) were homozygous and 23/114 (20%) heterozygous. Therefore, carrier frequency amongst non c124-2A>G homozygous cases was 34% (29/86), which does not differ significantly (p = 0.10) from the frequency of c124-2A>G carriers in the control cohort (211/829 = 26%). This suggests that the c124-2A>G mutation is the only common *RNF11* mutation involved in stunted growth in BBC.

To identify putative other loci involved in stunted growth, we genotyped the remaining 67 non c124-2A>G homozygous cases with a medium density 50 K SNP array (Illumina), and rescanned the genome as described before using only non c124-2A>G homozygous cases (86) and the same control cohort (275). As expected, there was no evidence for a residual effect of the *RNF11* locus. Neither was there any genome-wide significant evidence for other loci on any one of the 29 autosomes (Supporting Information S1)

## Discussion

We herein demonstrate that a loss-of-function mutation in the *RNF11* gene affects normal growth and disease resistance in calves. This is the first report of a phenotypic effect associated with *RNF11* mutations in any organism, including human and mouse [7].

We postulate that the increased disease susceptibility of homozygous c124-2A>G calves is related to the demonstrated role of RNF11 in feedback down-regulation of NF-κB by the A20 complex [7]. Indeed, the nine c124-2A>G homozygous calves that underwent necropsy were affected by extensive inflammation of the respiratory tract (eight) or by polyarthritis (one). Of note, A20 knock-out mice die prematurely from multi-organ inflammation [13]. The fact that only ∼1/3 of homozygous mutant calves died prematurely is compatible with a defect in the control or resolution of inflammation. External factors, including pathogens, may trigger an intendedly salutary innate and/or adaptive response, that evolves in pathogenic non-resolving inflammation [14].

The effects on growth may be secondary to hidden episodes of uncontrolled inflammation, as proposed for *A20*- and *ITCH*-deficient mice and human [13], [15], [16]. However, the fact that several of the surviving homozygous c124-2A>G calves appeared perfectly healthy upon clinical examination, suggest that growth retardation might be directly related to alternative functions of RNF11 as modulator of growth factor receptor signaling (particularly TGF-β and EGFR signaling) and transcriptional regulation [6]. It is also noteworthy, that *RNF11* has been found to be highly expressed in bone cells during osteogenesis [17].

Calf mortality is an economically important trait. It is generally considered highly complex and multifactorial, and its heritability is always very low. It is thus difficult to improve using conventional selection strategies. We herein demonstrate that genomic approaches may help dissect such complex phenotypes in sub-components including some with simple Mendelian determinism amenable to effective “marker assisted selection”. The situation uncovered in this work is reminiscent of bovine leukocyte deficiency (BLAD) in Holstein-Friesian [18], an immune deficiency resulting from CD18 deficiency and causing increased susceptibility to infection in young calves [19].

We provide suggestive evidence that the high incidence of the *RNF11* c124-2A>G mutation in BBC is not only due to drift, but may be due to the superiority of heterozygotes for unidentified selection criteria. Such a situation would be reminiscent of previously described pleiotropic effects on conformation of mutations in the gene encoding the *calcium release channel* (*CRC*) in pigs (causing malignant hyperthermia and porcine stress syndrome in homozygotes) [20] and in the *MRC2* gene in cattle (causing Crooked Tail Syndrome in homozygotes) [4], [5]. These examples illustrate some of the issues resulting from the selection of animals with extreme performances.

## Materials and Methods

### Ethics statement

Blood samples were collected from sires, cows and calves, by trained veterinarians following standard procedures and relevant national guidelines.

### Genotyping

Genomic DNA of cases was extracted from 350 µl of blood using the MagAttract DNA Blood Midi M48 Kit (Qiagen). Genomic DNA of controls was extracted from frozen semen using the MagAttract Mini M48 Kit (Qiagen). The 33 cases of the initial genome scan were genotyped using a custom-made 50 K SNP array [3]. The 67 cases of the second scan (excluding *RNF11* c124-2A>G homozygotes) were genotyped with the BovineSNP50 v2 DNA analysis BeadChip (Illumina). The 275 control sires were genotyped with the BovineHD BeadChip (Illumina). SNP genotyping was conducted using standard procedures at the GIGA genomics core facility.

### Genome-wide haplotype-based association studies

Phasing of the SNP genotypes and assignment of the haplotypes to a predetermined number of hidden haplotype states was conducted with PHASEBOOK [21]. Hidden haplotype state-based association analysis was conducted using GLASCOW (Zhang *et al.*, submitted for publication). GLASCOW uses generalized linear models and fits a random hidden haplotype state effect as well as a random polygenic effect to correct for population stratification. Locus-specific p-values were determined from 1,000 permutations assuming a gamma distribution of the used score test (Zhang *et al.*, submitted for publication). We applied a conservative Bonferonni correction assuming 50,000 independent tests to determine the genome-wide significance thresholds.

### Mutation scanning

Coding exons of positional candidate genes were amplified from genomic DNA of a homozygous case and a healthy control using standard procedures. The primers used for the *RNF11* gene are listed in the Supporting Information S1. PCR products were directly sequenced using the Big Dye terminator cycle sequencing kit (Applied Biosystem, Foster City, CA). Electrophoresis of purified sequencing reactions was performed on an ABI PRISM 3730 DNA analyzer (PE Applied Biosystems, Forster City, CA). Multiple sequence traces from affected and wild-type animals were aligned and compared using the Phred/Phrap/Consed package (www.genome.washington.edu).

### 5′ exonuclease diagnostic assay of the c124-2A>G *RNF11* mutation

A 5′ exonuclease assay was developed to genotype the c124-2A>G *RNF11* mutation, using 5′-AGG AAG AAA CAA AAG GAA AAC ATT ACC TAG A-3′ and 5′-TGT TGG ATG ATA GAC CGG AAC TG-3′ as PCR primers, and 5′-ACT TGT TCC **T**AA ATT TT-3′ (wild type *A* allele) and 5′-TTG TTC C**C**A AAT TTT-3′ (mutant *G* allele) as probes (Taqman, Applied Biosystems, Fosters City, CA). Reactions were carried out on an ABI7900HT instrument (Applied Biosystems, Fosters City, CA) using standard procedures.

### RT–PCR and cDNA sequencing

Total RNA from *RNF11* c124-2A>G *AA* and *GG* animals was extracted from lung, lymph nodes, spleen, skeletal muscle, thymus and trachea using standard procedures (Trizol, Invitrogen). After *DNase*-treatment (Turbo DNA-free, Ambion), cDNA was synthesized using the SuperScript III First-Strand Synthesis SuperMix (Invitrogen). A cDNA segment was amplified using two *RNF11* specific primers sets: one encompassing exon 2 with primers located in exon 1 and exon 3 (E1–E3) and one encompassing the exon1-exon2 boundary (E1–E2) (Supporting Information S1). PCR products were separated by electrophoresis on a 2% agarose gel containing 0.0001% of SYBR Safe DNA gel stain (Invitrogen) at 100 volts during 40 min and size was evaluated with SmartLadder 200 lanes (Eurogentec). The PCR products were directly sequenced as described above.

### Real-time quantitative RT–PCR

Total RNA from *RNF11* c124-2A>G *AA* and *GG* animals was extracted from lymph node, spleen as described above. After *DNase*-treatment (Turbo DNA-free, Ambion), 500 ng of total RNA was reverse transcribed in a final volume of 20 µl using SuperScript III First-Strand Synthesis SuperMix (Invitrogen). PCR reactions were performed in a final volume of 10 µl containing 4 µl of 5-fold diluted cDNA (corresponding to 100 ng of starting total RNA), 1X of ABsolute Blue QPCR SYBRE Green ROX Mix 2X (Thermo Fischer Scientific), 0.3 µM forward and reverse primers and nuclease free water. PCR reactions were performed on an ABI7900HT instrument (Applied Biosystems, Forster City, CA) under the following conditions: 10 min at 95°C followed by 40 cycles at 95°C for 15 sec and 60°C for 1 min. Two primers sets were used to test *RNF11* expression and three genes were included as candidate endogenous controls: (1) Beta-Actin (*ACTB*), (2) Ribosomal Protein Large P0 (*RPLP0*), (3) Tyr-3- & Trp-5-Monooxygenase Activation Protein Zeta (*YWHAZ*). The corresponding primer sequences are given in Supporting Information S1. A standard curve with a five point two-fold dilution series (total RNA = 100, 200, 400, 800 and 1600 ng from lymph node and spleen from a *AA* wild-type individual) for each *RNF11* primer set was used to determine the amplification efficiency. All sample/gene combinations were analyzed in triplicate. *ACTB* and *YWHAZ* genes were selected as endogenous controls using geNorm [22]. Normalized relative *RNF11* expression, for exon 2- and exon 3-containing transcripts, in the lymph node and the spleen of a wild-type *AA* and a mutant *GG* animal accounting for primer efficiency were computed using the qbase^plus^ software package (Biogazelle) [23].

### Estimating the effect of carrier status for the *RNF11* c124-2A>G mutation on agronomically important traits measured in the field

The effect of the sire's *RNF11* c124-2A>G genotype on non-return rate (NRR) of its mates was estimated using a mixed model including sire's *RNF11* genotype (fixed), year and month at insemination (fixed), mate's herd (random), individual animal effect of the offspring (random) and error. NRR are computed from the AI information collected by inseminators working with the Association Wallonne de l'Elevage (AWE; http://www.awenet.be/) at seven time-points after AI. The analysis was performed on 479,674 cows mated to 340 AI sires.

The effect of the sire's *RNF11* c124-2A>G genotype on the rate of mortality, morbidity and culling of its offspring was estimated using a mixed model including sire's *RNF11* genotype (fixed), calf's gender (fixed), year and month of calf's birth (fixed), mate's parity (fixed), calf's *in utero* position (fixed; forward or backward), calf's herd (random), individual animal effect of the calf (random), and error. The corresponding phenotypes are collected by AWE technicians visiting farms, for (i) newborn calves, and (ii) calves having reached the age of 14 months since last visit. The number of records for newborn offspring was 317,350 from 332 AI sires, and for 14 month-old offspring was 126,098 from 288 AI sires.

The effect of the sire's *RNF11* c124-2A>G genotype on its own zootechnical performances was estimated using a mixed model including sire's *RNF11* genotype (fixed), sire's *MRC2* genotype (fixed) [4], [5], year and month at scoring (fixed), sire's body condition at scoring (fixed), sire's age at scoring (quadratic regression), individual animal effect for the sires (random) and error [24]. Zootechnical performances of AI sires are recorded between 15 and 56 months of age as 22 linear scores (0–50 score) that are summarized as indexes evaluating size, muscularity, meaty type and general appearance [12]. Three hundred and eleven sires were used in this analysis.

The effect of the sire's *RNF11* c124-2A>G genotype on the zootechnical performances if its offspring was estimated using a mixed model including sire's *RNF11* genotype (fixed), sire's *MRC2* genotype (fixed) [4], [5], offspring's gender (fixed), year and month at scoring (fixed), offspring's body condition at scoring (fixed), offspring's age at scoring (quadratic regression), offspring's herd (random), individual animal effect for the offspring (random) and error [24]. The first data set corresponded to the same five global scores (cfr. sire's own performances) measured on 92,475 36-month-old daughters of 306 sires by AWE technicians. The second data set corresponded to weight (Kg), size (cm) and conformation (1–9 score) measured on 95,045 14-month-old offspring of 315 sires.

Covariances between random individual animal effects were assumed to be proportionate to twice the kinship coefficient computed from known genealogies. Variance components and fixed effects were computed using MTDFREML [25].

## Supporting Information

Supporting Information S1 Supporting figures and tables.
(PDF)
