# Supplementary material for: A Splice Site Variant in the Bovine RNF11 Gene Compromises Growth and Regulation of the Inflammatory Response
Source: PLoS Genet. 2012 Mar 15;8(3):e1002581. doi: 10.1371/journal.pgen.1002581 (PMC3305398; doi:10.1371/journal.pgen.1002581)
Supplement: Supporting Information S1 — Supporting figures and tables. (PDF) [file pgen.1002581.s001.pdf]

Supporting Information S1

A splice site variant in the bovine *RNF11* gene compromises growth and regulation of the inflammatory response.

Arnaud Sartelet[1], Tom Druet[1], Charles Michaux[2], Corinne Fasquelle[1], Sarah Géron[1], Nico Tamma[1], Zhiyan Zhang[1], Wouter Coppieters[1], Michel Georges[1], Carole Charlier[1§].

[1]Unit of Animal Genomics, GIGA-R & Department of Animal Sciences, Faculty of Veterinary Medicine, University of Liège, Belgium.

[2]Unit of Bioinformatics, Department of Animal Sciences, Faculty of Veterinary Medicine, University of Liège, Belgium.

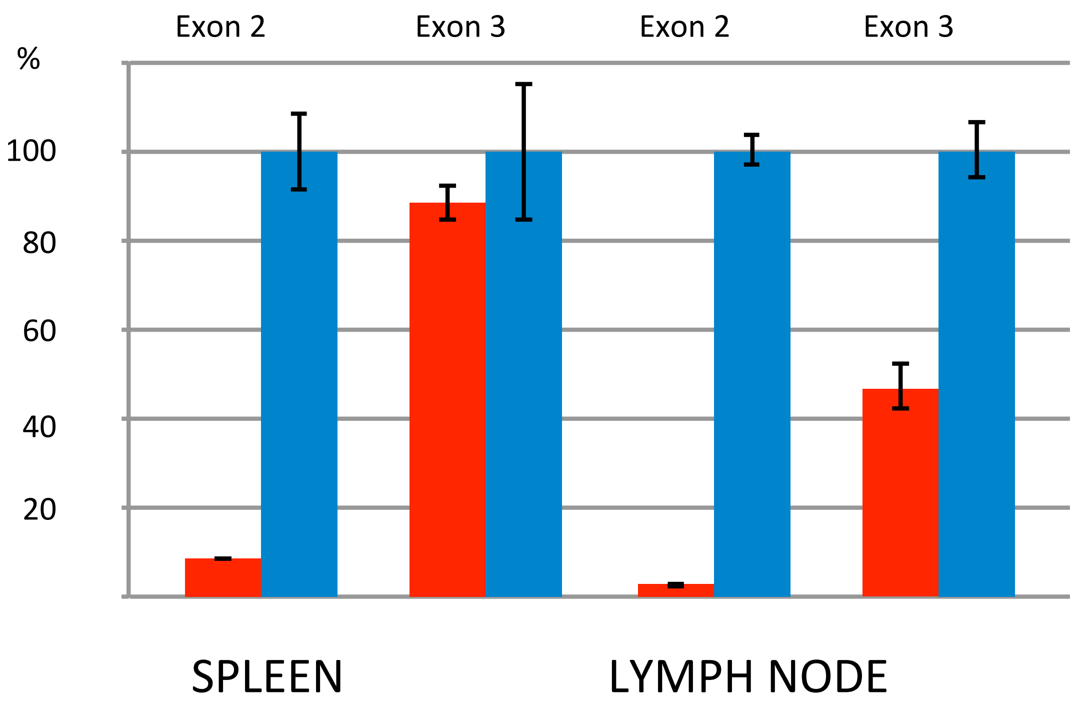

Supplementary figure 1. Comparing *RNF11* exon 2 and exon 3 transcript levels in the spleen (left) and in the mesenteric lymph node (right) of *AA* wild-type (blue columns) and *GG* mutant animals (red columns). Error bars correspond to standard errors over three replicates per sample.

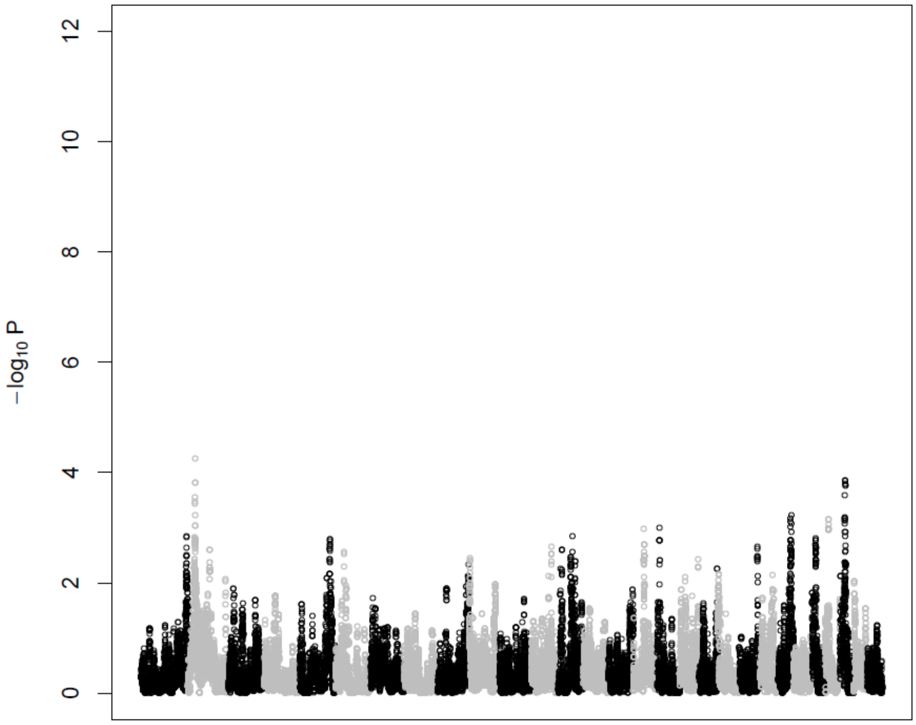

Supplementary figure 2. Lack of evidence for other growth stunting loci. Manhattan plot for the second haplotype-based genome-wide association study for 67 stunted-growth cases (excluding c124-A>G homozygous) using a model with 20 ancestral haplotypes (20 hidden haplotypes states).

Supplementary table 1: Effect of carrier status of AI sires on non return rate of mates and on the rate of mortality, morbidity and culling of offspring.

		**Contrast ($AA - AG$)**	**p-value**
**NON RETURN RATE (Days after AI)**	28	0.0067	0.7266
	56	-0.0009	0.9602
	84	0.0053	0.7796
	112	0.0060	0.7492
	150	0.0082	0.6602
	200	0.0053	0.7796
	280	0.0065	0.7266
**MORTALITY**	0 – 3 months	-0.0019	0.9761
	0 – 14 months	-0.0128	0.8966
	1 – 14 months	-0.0101	0.9204
	6 – 14 months	-0.0079	0.9363
**MORTALITY + MORBIDITY + CULLING**	0 – 14 months	-0.0108	0.9125
	1 – 14 months	-0.0106	0.9046

Supplementary table 2: Necropsy findings and genotype of the deceased calves in the prospective study.

CALF ID	AGE (months)	NECROPSY	GENOTYPE
4322	6	FRACTURE	*AG*
4334	6	PNEUMONIA	*GG*
4296	2	PNEUMONIA + MENINGITIS	*GG*
4477	3	PNEUMONIA + RUMINAL APLASIA	*GG*
4430	4	PNEUMONIA	*GG*
4272	< 1	PNEUMONIA + JEJUNAL APLASIA	*GG*
3806	< 1	LARYNGOTRACHEITIS	*GG*
4380	2	PNEUMONIA + DIARRHEA	*GG*
4113	3	POLYARTHRITIS	*GG*
4548	2	MENINGITIS	*GG*

Supplementary table 3: Effect of carrier status of sires on own zootechnical performances and that of their offspring.

		**Contrast (*AA - AG)***	**p-value**	**Carrier characteristics**
**Performance AI sires (36 months)**	Size (cm)	-1.15	0.2766	
	Muscularity	0.28	0.4128	
	Meaty type	0.39	0.1780	
	General appearance	0.45	0.0071	Worse
**Performance Daughters (36 months)**	Size (cm)	0.26	0.0777	
	Muscularity	-0.36	0.0025	Increased muscularity
	Meaty type	-0.23	0.0103	Increased muscularity
	General appearance	-0.15	0.01006	Better
**Progeny test (14 months)**	Size (cm)	0.26	0.1372	
	Conformation	-0.13	0.0005	Increased muscularity
	Weight (kg)	3.59	0.0152	Lighter

Supplementary table 4: Primer pairs for the *RNF11* gene.

Name	Primer sequence (5’-3’)	Gene part	Size (bp)
gUP1	GATGTAGGAGGATTGGAAAGTG	Exon 1 (ATG)	395 bp
gDN1	CGTGAAGCAGGGAGATGTCATC		
gUP2	CTTTCTTCCTCCCCCAGATCAC	Exon 1 (ATG)	352 bp
gDN2	TTAAAGGTTTCCAAAGTTCAAG		
gUP3	AGAAACAAAAGGAAAACATTAC	Exon 2	506 bp
gDN3	ATGATCAAGTGTGAATAATGTG		
gUP4	GTGATAGAATGACAGGAAGCCG	Exon 3	1292 bp
gDN4	TTGTCCCTTCCAGTGTCCTTTC		
gUP5	TATCGTTGGGGCTGGCTCTATG	Exon 3 (STOP)	1313 bp
gDN5	TGATATTGTGAACTACTGTCTG		

Supplementary table 5: Primer pairs for the detection of exon 2 skipping and alternative splicing.

Name	Primer sequence (5’-3’)	Size (bp)	
cUP_E1	CCCTGCTTCACGAGTCTCAGTC	231 bp	360 bp
cDN_E2	CATCTCTTCCAGGGTCATAAAC		
cDN_E3	AGGATCTCATCAACCAGTCATC		

Supplementary table 6: Quantitative RT-PCR primers .

Gene	Forward primer	Reverse primer	Size (bp)
RNF11_E2	GTTCCGGTCTATCATCCAAC	TCTCTTCCAGGGTCATAAAC	134 bp
RNF11_E3	GTATAGATGACTGGTTGATGAG	TTCCTAATCCCTGGCTCTTTGG	182 bp
ACTB	TCGCGGACAGGATGCAGAAAGA	GCTGATCCACATCTGCTGGAA	149 bp
YWHAZ	GCATCCCACAGACTATTTCC	GCAAAGACAATGACAGACCA	120 bp
RPLP0	TGGGCAAGAACACGATGATG	TGAGGTCCTCCTTGGTGAACA	123 bp
